# Complete genome sequence of *Rhizobium leguminosarum* bv *trifolii* strain WSM2304, an effective microsymbiont of the South American clover *Trifolium polymorphum.*

**DOI:** 10.4056/sigs.44642

**Published:** 2010-02-28

**Authors:** Wayne Reeve, Graham O’Hara, Patrick Chain, Julie Ardley, Lambert Bräu, Kemanthi Nandesena, Ravi Tiwari, Stephanie Malfatti, Hajnalka Kiss, Alla Lapidus, Alex Copeland, Matt Nolan, Miriam Land, Natalia Ivanova, Konstantinos Mavromatis, Victor Markowitz, Nikos Kyrpides, Vanessa Melino, Matthew Denton, Ron Yates, John Howieson

**Affiliations:** 1 Centre for *Rhizobium* Studies, Murdoch University, Western Australia, Australia; 2 DOE Joint Genome Institute, Walnut Creek, California, USA; 3 Lawrence Livermore National Laboratory, Livermore, California, USA; 4 Oak Ridge National Laboratory, Oak Ridge, Tennessee, USA; 5 Biological Data Management and Technology Center, Lawrence Berkeley National Laboratory, Berkeley, California, USA; 6 Department of Primary Industries, Victoria, Australia; 7 Department of Agriculture and Food, Western Australia, Australia

**Keywords:** microsymbiont, non-pathogenic, aerobic, Gram-negative rod, root-nodule bacteria, nitrogen fixation, *Alphaproteobacteria*

## Abstract

*Rhizobium leguminosarum* bv *trifolii* is the effective nitrogen fixing microsymbiont of a diverse range of annual and perennial *Trifolium* (clover) species. Strain WSM2304 is an aerobic, motile, non-spore forming, Gram-negative rod, isolated from *Trifolium polymorphum* in Uruguay in 1998. This microsymbiont predominated in the perennial grasslands of Glencoe Research Station, in Uruguay, to competitively nodulate its host, and fix atmospheric nitrogen. Here we describe the basic features of WSM2304, together with the complete genome sequence, and annotation. This is the first completed genome sequence for a nitrogen fixing microsymbiont of a clover species from the American center of origin. We reveal that its genome size is 6,872,702 bp encoding 6,643 protein-coding genes and 62 RNA only encoding genes. This multipartite genome was found to contain 5 distinct replicons; a chromosome of size 4,537,948 bp and four circular plasmids of size 1,266,105 bp, 501,946 bp, 308,747 bp and 257,956 bp.

## Introduction

Since ancient times, crop fields have been regularly rotated with legumes, and this continues in the modern world because of the recognition that the productivity of agricultural systems is nitrogen dependent [1]. Legumes may redress nitrogen deficiency through the fixation of atmospheric nitrogen by rhizobia in root nodules [2]. Today, despite the ready availability of nitrogen-fertilizer manufactured through the Haber-Bosch process, globally in excess of 400 million ha of agricultural land are sustained by nitrogen derived from forage legumes [3]. These forages are grown for animal feed, for rotation with cereal crops, as disease breaks or as cover crops for tree plantations. Amongst the forage legumes, the *Trifolium* spp. (clovers) are acknowledged as one of the most important genera, with 237 species distributed across the temperate and sub-tropical regions of North and South America, Europe, Africa and Australasia [4].

These clovers are nodulated by *R. leguminosarum* bv *trifolii*, which is one of the most exploited species of root-nodule bacteria in world agriculture. However, because clovers are geographically widely distributed, and phenologically variable (they may be either annual [e.g. *T. subterraneum*] or perennial [e.g. *T. pratense, T. raepens* and *T. polymorphum*]), it is rare that a single strain of *R. leguminosarum* bv *trifolii* can effectively fix nitrogen across a wide diversity of clovers, especially those from different geographical and phenological backgrounds [5].

*Rhizobium leguminosarum* bv *trifolii* strain WSM2304 was isolated from a nodule recovered from the roots of the perennial clover *Trifolium polymorphum* growing at Glencoe Research Station near Tacuarembó, Uruguay in December 1998. WSM2304 is of particular interest because it is a highly effective microsymbiont of a perennial clover of South American origin, has a narrow, specialized host range for nitrogen fixation [5], and is highly competitive for nodulation of *T. polymorphum* in the acid, infertile soils of Uruguay [6]. WSM2304 has also been implicated in host mediated selection for an effective microsymbiont under competitive conditions for nodulation [7].

Here we present a summary classification and a set of features for *R. leguminosarum* bv *trifolii* strain WSM2304 (Table 1), together with the description of the complete genome sequence and annotation.

**Table 1:** Classification and general features of *R. leguminosarum* bv *trifolii* WSM2304 in accordance with the MIGS recommendations [8].

MIGS ID	Property	Term	Evidence code
	Classification	Domain *Bacteria*	TAS [5-7,9]
		Phylum *Proteobacteria*	TAS [5-7,10]
		Class *Alphaproteobacteria*	TAS [5-7,11,12]
		Order *Rhizobiales*	TAS [5-7,11,13]
		Family *Rhizobiaceae*	TAS [5-7,14]
		Genus *Rhizobium*	TAS [5-7,14-18]
		Species *Rhizobium leguminosarum* bv *trifolii*	TAS [5-7,14,16,18,19]
		Strain WSM2304	
	Gram stain	negative	TAS [20]
	Cell shape	rod	TAS [20]
	Motility	motile	TAS [20]
	Sporulation	non-sporulating	TAS [20]
	Temperature range	mesophile	TAS [20]
	Optimum temperature	28°C	TAS [20]
	Salinity	unknown	TAS [20]
MIGS-22	Oxygen requirement	aerobic	TAS [20]
	Carbon source	glucose, mannitol	TAS [5-7]
	Energy source	chemoheterotroph	TAS [20]
MIGS-6	Habitat	Soil, root nodule, host	TAS [5-7]
MIGS-15	Biotic relationship	Free living, Symbiotic	TAS [5-7]
MIGS-14	Pathogenicity	none	TAS [20]
	Biosafety level	1	TAS [21]
	Isolation	*Trifolium polymorphum* root nodule	TAS [22]
MIGS-4	Geographic location	Glencoe Research Station, INIA, Uruguay	TAS [22]
MIGS-5	Sample collection time	December 1^st^, 1998	TAS [22]
MIGS-4.1 MIGS-4.2	Latitude – Longitude	-56 -31.41	TAS [22]
MIGS-4.3	Depth	5cm soil depth	NAS [2]
MIGS-4.4	Altitude	130m	TAS [22]

Evidence codes - IDA: Inferred from Direct Assay (first time in publication); TAS: Traceable Author Statement (i.e., a direct report exists in the literature); NAS: Non-traceable Author Statement (i.e., not directly observed for the living, isolated sample, but based on a generally accepted property for the species, or anecdotal evidence). These evidence codes are from the Gene Ontology project [23]. If the evidence code is IDA, then the property was directly observed for a living isolate by one of the authors or an expert mentioned in the acknowledgements

## Classification and features

**Figure 1:**
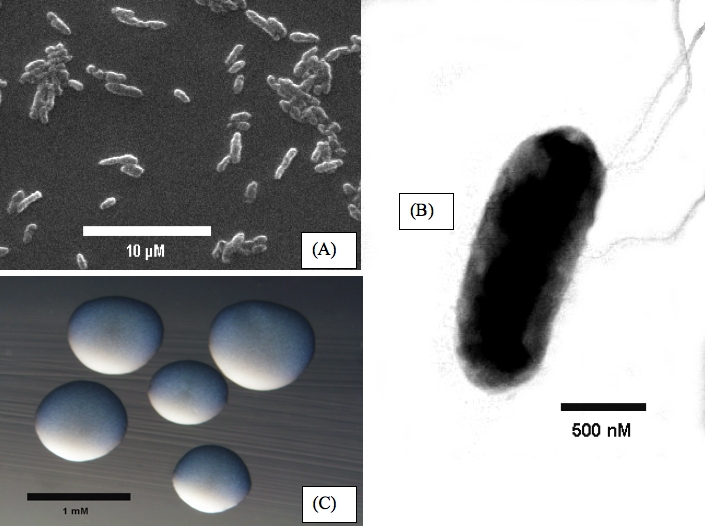
Images of *R. leguminosarum* bv *trifolii* strain WSM2304 using scanning (A) and transmission electron microscopy (B). The appearance of colony morphology on solid media (C).

*R. leguminosarum* bv *trifolii* strain WSM2304 is a motile, Gram-negative, non-spore-forming rod (Figure 1 A and B) in the *Rhizobiaceae* family of the class *Alphaproteobacteria* that forms mildly mucoid colonies (Figure 1 C) on solid media [24]. It has a mean generation time of 3.5 h in rich medium at the optimal growth temperature of 28°C [7].

**Figure 2:**
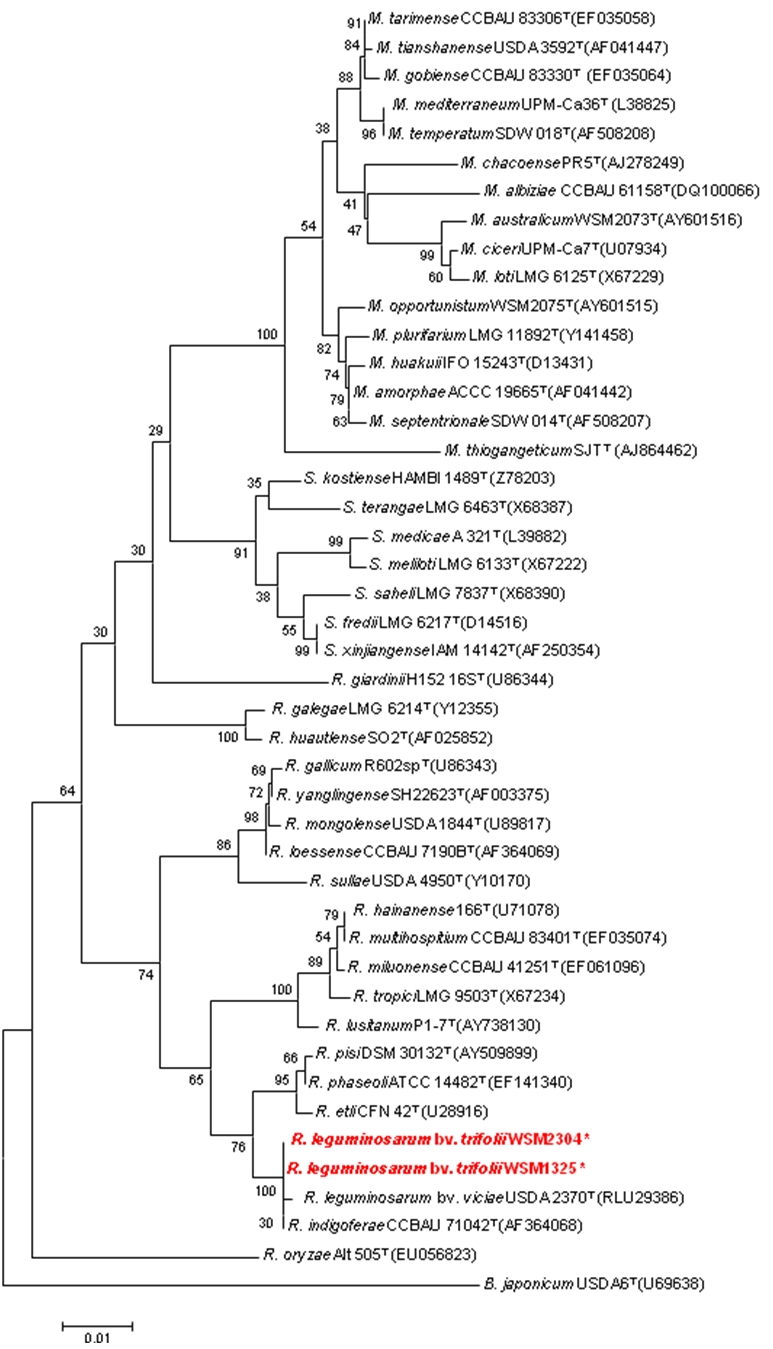
Phylogenetic tree showing the relationships of *R. leguminosarum* bv *trifolii* strain WSM2304 with the type strains of *Rhizobiaceae* based on aligned sequences of the 16S rRNA gene (1,440 bp internal region). All sites were informative and there were no gap-containing sites. Phylogenetic analyses were performed using MEGA, version 3.1 [25]. Kimura two-parameter distances were derived from the aligned sequences [26] and a bootstrap analysis [27] as performed with 500 replicates in order to construct a consensus unrooted tree using the neighbor-joining method [28] for each gene alignment separately. The genera in this tree include *Bradyrhizobium* (B.), *Mesorhizobium* (M), *Rhizobium* (R); *Ensifer* (*Sinorhizobium*) (S). Type strains are indicated with a superscript T. Strains with a genome sequencing project registered in GOLD [22] are in bold red print. Published genomes are designated with an asterisk.

Figure 2 shows the phylogenetic neighborhood of *R. leguminosarum* bv *trifolii* strain WSM2304 in a 16S rRNA-based tree. An intragenic fragment of 1,440 bp was chosen since the 16S rRNA gene has not been completely sequenced in many type strains. A comparison of the entire 16S rRNA gene of WSM2304 to completely sequenced 16S rRNA genes of other rhizobia revealed 100% gene sequence identity with *R. leguminosarum* bv *trifolii* strain WSM1325 but a 1 bp difference from the 16S rRNA gene of *R. leguminosarum* bv *viciae* strain 3841.

### Symbiotaxonomy

*R. leguminosarum* bv *trifolii* WSM2304 nodulates (Nod^+^) and fixes nitrogen effectively (Fix^+^) with the South American perennial clover *T. polymorphum* [5]. WSM2304 is Nod^+^, Fix^-^ with Mediterranean annual clovers *T. subterraneum* and *T. glanduliferum*, in contrast to *R. leguminosarum* bv *trifolii* WSM1325 [5,29]. When inoculated onto perennial clovers of either North American or Mediterranean origin WSM2304 is variably Nod^+^, but always Fix^-^ [5,6,30]. Under conditions of competitive nodulation, WSM2304 may preferentially nodulate *T. polymorphum* even when outnumbered 100:1 by WSM1325 [7].

## Genome sequencing and annotation information

### Genome project history

This organism was selected for sequencing on the basis of its environmental and agricultural relevance to issues in global carbon cycling, alternative energy production, and biogeochemical importance, and is part of the Community Sequencing Program at the Department of Energy Joint Genome Institute (JGI) for projects of relevance to DOE missions. The genome project is deposited in the Genomes OnLine Database [22] and the complete genome sequence in GenBank. Sequencing, finishing and annotation were performed by the DOE Joint Genome Institute (JGI). A summary of the project information is shown in Table 2 and sequence data statistics from the trace archive for this project are presented in Table 3.

**Table 2:** Genome sequencing project information for *R. leguminosarum* bv *trifolii* WSM2304.

MIGS ID	Property	Term
MIGS-31	Finishing quality	Finished
MIGS-28	Libraries used	Four genomic libraries: three Sanger libraries;1-2 kb pTH1522, 6-8 kb pMCL200, fosmid pcc1Fos and one 454 pyrosequencing standard library
MIGS-29	Sequencing platforms	ABI3730xl, 454 GS FLX
MIGS-31.2	Sequencing coverage	21.3 x Sanger; 10.1 x Pyrosequencing
MIGS-30	Assemblers	Newbler version 1.1.02.15, Phrap
MIGS-32	Gene calling method	Prodigal
	Genbank ID	CP001191 (Chromosome)^a^ CP001192 (pRLG201)^b^ CP001193 (pRLG202)^c^ CP001194 (pRLG204)^d^ CP001195 (pRLG205)^e^
	Genbank Date of Release	16-OCTOBER-2008
	GOLD ID	Gc00870^f^
	NCBI project ID	20179
	Database: IMG	643348569 ^g^
	Project relevance	Symbiotic nitrogen fixation, agriculture

^a^ http://www.ncbi.nlm.nih.gov/nuccore/209533368

^b^ http://www.ncbi.nlm.nih.gov/nuccore/209537694

^c^ http://www.ncbi.nlm.nih.gov/nuccore/209538856

^d^ http://www.ncbi.nlm.nih.gov/nuccore/209539307

^e^ http://www.ncbi.nlm.nih.gov/nuccore/209539531

^f^ http://genomesonline.org/GOLD_CARDS/Gc00870.html

^g^ http://img.jgi.doe.gov/cgi-bin/pub/main.cgi?section=TaxonDetail&page=taxonDetail&taxon_oid=64173348569

**Table 3:** Production sequence for the finished genome of *R. leguminosarum* bv *trifolii* WSM2304, JGI project 4024175

**Vector/Type**	**Library id**	**Insert size (kb)**	**Reads**	**Mb**	**q20 (Mb)**
pMCL200	FHOO	7.0 ± 0.9	74,398	66.6	51.6
pcc1Fos	FHTU	36 ± 3.4	15,776	11.8	7.8
pTH1522	FNNZ	1.8 ± 0.3	79,386	68.6	53.5
454-std	FHTW	NA	719,338	69.9	NA

### Growth conditions and DNA isolation

*R. leguminosarum* bv *trifolii* WSM2304 was grown to mid logarithmic phase in TY medium (a rich medium) [31] on a gyratory shaker at 28°C. DNA was isolated from 60 ml of cells using a CTAB (Cetyl trimethylammonium bromide) bacterial genomic DNA isolation method (http://my.jgi.doe.gov/general/index.html).

### Genome sequencing and assembly

The genome was sequenced using a combination of Sanger and 454 sequencing platforms. All general aspects of library construction and sequencing performed at the JGI can be found at the JGI website (http://www.jgi.doe.gov/). 454 Pyrosequencing reads were assembled using the Newbler assembler version 1.1.02.15 (Roche). Large Newbler contigs were broken into 5,676 fragments of 1,500 bp with 100 bp overlap and entered into the assembly as pseudo-reads. The sequences were assigned quality scores based on Newbler consensus q-scores with modifications to account for overlap redundancy and to adjust inflated q-scores. A hybrid 454/Sanger assembly was made using the phrap assembler. Possible mis-assemblies were corrected and gaps between contigs were closed by custom primer walks from sub-clones or PCR products. A total of 1,826 Sanger finishing reads were produced. Illumina reads were used to improve the final consensus quality using an in-house developed tool (the Polisher). The final assembly consists of 168,617 Sanger reads in addition to 5,663 454 pseudo reads. The error rate of the completed genome sequence is less than 1 in 100,000. Together all sequence types provided about 31.4× coverage of the genome.

### Genome annotation

Genes were identified using Prodigal [32] as part of the Oak Ridge National Laboratory genome annotation pipeline, followed by a round of manual curation using the JGI GenePRIMP pipeline [33]. The predicted CDSs were translated and used to search the National Center for Biotechnology Information (NCBI) nonredundant database, UniProt, TIGRFam, Pfam, PRIAM, KEGG, COG, and InterPro databases. Additional gene prediction analyses and functional annotation were performed within the Integrated Microbial Genomes  platform (http://img.jgi.doe.gov/er) [34].

## Genome properties

**Figure 3:**
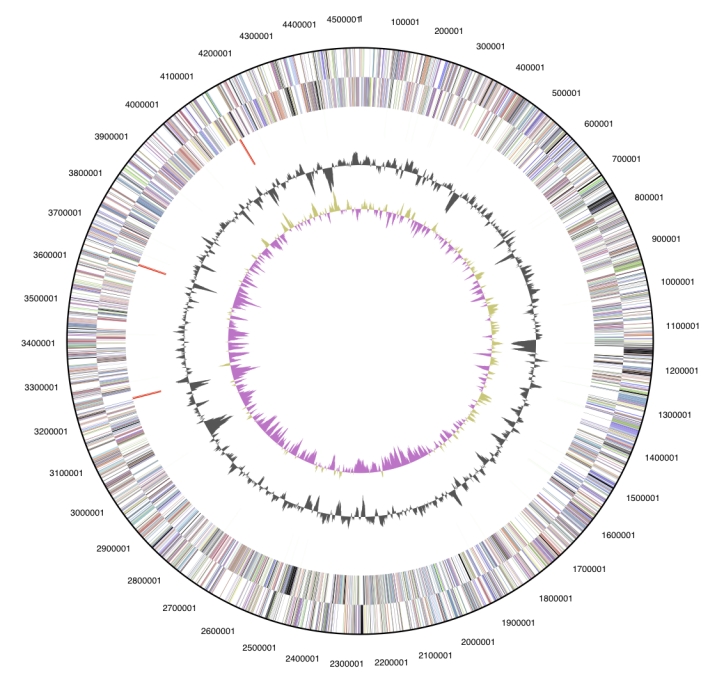
Graphical circular map of the chromosome of *R. leguminosarum* bv *trifolii* WSM2304. From outside to the center: Genes on forward strand (color by COG categories as denoted by the IMG platform), Genes on reverse strand (color by COG categories), RNA genes (tRNAs green, sRNAs red, other RNAs black), GC content, GC skew. Chromosome is not drawn to scale relative to the plasmids in Figure 4.

**Figure 4:**
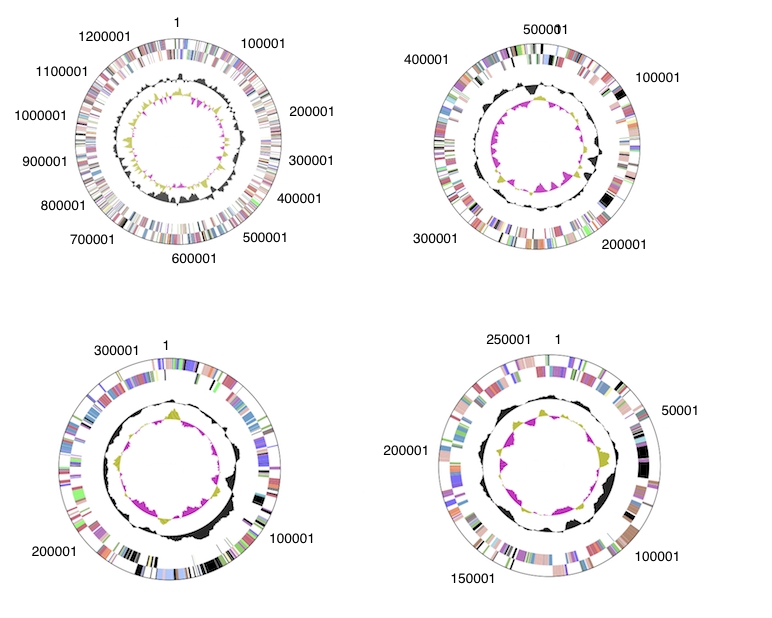
Graphical circular map of the plasmids of *R. leguminosarum* bv *trifolii* WSM2304. From outside to the center: Genes on forward strand (color by COG categories as denoted by the IMG platform), Genes on reverse strand (color by COG categories), RNA genes (tRNAs green, sRNAs red, other RNAs black), GC content, GC skew. Plasmids pRLG201, pRLG202, pRLG203 and pRLG204 are not drawn to scale relative to each other or to the chromosome in Figure 3.

The genome is 6,872,702 bp long with a 61.18% GC content, (Table 4) and comprised of 5 replicons; 1 circular chromosome of size 4,537,948 bp (Figure 3) and 4 circular plasmids of size 4,537,948, 1,266,105, 501,946, 308,747 and 257,956 bp (Figure 4). Of the 6,643 genes predicted, 6,581 were protein coding genes, and 62 RNA only encoding genes. In addition, 166 pseudogenes were identified. The majority of the genes (72.44%) were assigned a putative function whilst the remaining ones were annotated as hypothetical proteins. The distribution of genes into COGs functional categories is presented in Table 5.

**Table 4:** Genome Statistics for *R. leguminosarum* bv *trifolii* WSM2304.

**Attribute**	**Value**	**% of Total**
Genome size (bp)	6,872,702	100.00%
DNA coding region (bp)	6,053,973	88.09%
DNA G+C content (bp)	4,204,577	61.18%
Number of replicons	5	100.00%
Extrachromosomal elements	4	80.00%
Total genes	6,643	100.00%
RNA coding genes	62	0.93%
rRNA operons	3	
Protein-coding genes	6,581	99.07%
Pseudo genes	166	2.49%
Genes with function prediction	4,812	72.44%
Genes in paralog clusters	4,104	61.78%
Genes assigned to COGs	5,105	76.85%
Genes assigned Pfam domains	5,149	77.51%
Genes with signal peptides	2,247	33.83%
Genes with transmembrane helices	1,495	22.50%
CRISPR repeats	0	

**Table 5:** The number of predicted protein-coding genes of *R. leguminosarum* bv *trifolii* WSM2304 associated with the 21 general COG functional categories.

**Code**	**value**	**%age**		**Description**
J	194		2.95	Translation, ribosomal structure and biogenesis
A	0		0.00	RNA processing and modification
K	558		8.48	Transcription
L	164		2.49	Replication, recombination and repair
B	2		0.03	Chromatin structure and dynamics
D	38		0.58	Cell cycle control, mitosis and meiosis
Y	0		0.00	Nuclear structure
V	66		1.00	Defense mechanisms
T	317		4.82	Signal transduction mechanisms
M	309		4.70	Cell wall/membrane biogenesis
N	91		1.38	Cell motility
Z	0		0.00	Cytoskeleton
W	0		0.00	Extracellular structures
U	88		1.34	Intracellular trafficking and secretion
O	165		2.51	Posttranslational modification, protein turnover, chaperones
C	314		4.77	Energy production and conversion
G	599		9.10	Carbohydrate transport and metabolism
E	687		10.44	Amino acid transport and metabolism
F	109		1.66	Nucleotide transport and metabolism
H	180		2.74	Coenzyme transport and metabolism
I	238		3.62	Lipid transport and metabolism
P	278		4.22	Inorganic ion transport and metabolism
Q	156		2.37	Secondary metabolites biosynthesis, transport and catabolism
R	710		10.79	General function prediction only
S	532		8.08	Function unknown
-	1,476		22.43	Not in COGs
